# Fracture risk assessment in long-term care*:* a survey of long-term care physicians

**DOI:** 10.1186/1471-2318-13-109

**Published:** 2013-10-18

**Authors:** Michelle Wall, Lynne Lohfeld, Lora Giangregorio, George Ioannidis, Courtney C Kennedy, Andrea Moser, Alexandra Papaioannou, Suzanne N Morin

**Affiliations:** 1 McGill University Health Center Research Institute, Montreal, Canada; 2 McMaster University, Hamilton, Canada; 3 University of Waterloo, Waterloo, Canada; 4 University of Toronto, Toronto, Canada; 5 Department of Medicine, McGill University, Montreal, Canada

**Keywords:** Fractures, Osteoporosis, Fracture risk assessment, Long-term care, Survey

## Abstract

**Background:**

The majority of frail elderly who live in long-term care (LTC) are not treated for osteoporosis despite their high risk for fragility fractures. Clinical Practice Guidelines for the diagnosis and management of osteoporosis provide guidance for the management of individuals 50 years and older at risk for fractures, however, they cannot benefit LTC residents if physicians perceive barriers to their application. Our objectives are to explore current practices to fracture risk assessment by LTC physicians and describe barriers to applying the recently published Osteoporosis Canada practice guidelines for fracture assessment and prevention in LTC.

**Methods:**

A cross-sectional survey was conducted with the Ontario Long-Term Care Physicians Association using an online questionnaire. The survey included questions that addressed members’ attitudes, knowledge, and behaviour with respect to fracture risk assessment in LTC. Closed-ended responses were analyzed using descriptive statistics and thematic framework analysis for open-ended responses.

**Results:**

We contacted 347 LTC physicians; 25% submitted completed surveys (81% men, mean age 60 (Standard Deviation [SD] 11) years, average 32 [SD 11] years in practice). Of the surveyed physicians, 87% considered prevention of fragility fractures to be important, but a minority (34%) reported using validated fracture risk assessment tools, while 33% did not use any. Clinical risk factors recommended by the OC guidelines for assessing fracture risk considered applicable included; glucocorticoid use (99%), fall history (93%), age (92%), and fracture history (91%). Recommended clinical measurements considered applicable included: weight (84%), thyroid-stimulating hormone (78%) and creatinine (73%) measurements, height (61%), and Get-Up-and-Go test (60%). Perceived barriers to assessing fracture risk included difficulty acquiring necessary information, lack of access to tests (bone mineral density, x-rays) or obtaining medical history; resource constraints, and a sentiment that assessing fracture risk is futile in this population because of short life expectancy and polypharmacy.

**Conclusion:**

Perceived barriers to fracture risk assessment and osteoporosis management in LTC have not changed recently, contributing in part to the ongoing care gap in osteoporosis management. Our findings highlight the importance to adapt guidelines to be applicable to the LTC environment, and to develop partnerships with stakeholders to facilitate their use in clinical practice.

## Background

Osteoporosis is a common disease characterized by low bone mass and increased risk for fractures [1]. Osteoporosis-related fractures are strongly associated with recurrent fractures, and are responsible for lasting disability, morbidity and excess mortality [2-6]. In Canada, the incidence of osteoporosis-related fractures is 7.2 and 15. 3/1000 person-years for men and for women over the age of 50 years, and the cost to the healthcare system has been estimated to be up to $3.9 billion annually [7].

In recent years, the focus of osteoporosis management has shifted from the treatment of low bone mineral density (BMD) to an integrated approach of fracture risk reduction through recognition of important clinical risk factors. In 2010, Osteoporosis Canada (OC) published evidence-based clinical practice guidelines for the diagnosis and management of osteoporosis that reflect the new approach to risk assessment. The guidelines strongly recommend the use of the validated fracture risk assessment tools FRAX or CAROC (Canadian Association of Radiologists and Osteoporosis Canada). These tools evaluate the risk of osteoporosis-related fractures based on individual risk factors and are appropriate for use in clinical practice [8]. Both tools are country- and sex- specific, and based on sets of risk factors that include age, BMD of the hip (FRAX, but not CAROC, can also provide a score in the absence of BMD measurement), prevalent fragility fractures, use of glucocorticoids and others, and predict the 10-year probability of major osteoporotic fractures (hip, spine, distal forearm and humerus). Results obtained from the FRAX or CAROC predictor tools are used to guide treatment so that pharmacotherapy is recommended for patients with an elevated 10-year probability of major fractures. Many countries have also recently reviewed and updated their clinical practices guidelines using a similar framework [9]. Knowledge may not benefit patients unless it is disseminated and translated to healthcare providers, patients and policy makers in a form they can use. Attention must be devoted to adapt this knowledge to the context and its stakeholders [10].

Elderly residents of long-term care (LTC) facilities have a higher prevalence of osteoporosis and higher rates of fracture morbidity and mortality following fractures than their age- and sex-matched community-dwelling counterparts [11]. Eighty-five percent of LTC residents are reported to have osteoporosis and 40% of all hip fractures occur in this population [12,13]. Nevertheless, the majority of these frail individuals are not treated for osteoporosis [14]. To better understand this care gap, and to inform the development of practice guidelines specific to the LTC setting, we conducted a survey to collect information and opinions on fracture risk assessment from frontline physicians in the LTC environment. Our objectives were to explore which elements of the OC guidelines’ recommendations are perceived to be applicable in the LTC setting, to inquire about current practices for fracture risk assessment by LTC physicians and to describe barriers to applying the OC guidelines for fracture assessment and prevention in LTC.

## Methods

### Sample population

Using electronic mail, we contacted all 347 physician-members of the Ontario Long-term Care Physicians Association to complete a self-administered on-line survey. The survey was available from May to June 2012; reminders were sent out twice, 2 weeks apart. This group was chosen for their interest and expertise in LTC and because of previous participation in surveys of similar design [15,16]. Ethics approval was granted from the Hamilton Integrated Research Ethics Board; participants gave consent at the time of survey completion.

### Survey design

The questionnaire (11 items, including 3 open-ended questions) was developed by a multidisciplinary panel based on Dillman’s principles of web-based questionnaire designs [17]. These principles provide guidance on the construction of self-administered questionnaires so that their structure and the technical interface encourage responders to connect and respond. Question generation was informed by three sources: literature review, input from clinicians with experience in LTC and the authors’ expertise.

Questionnaire content and face validity were reviewed by the team of authors who included clinicians and researchers with expertise in LTC, osteoporosis management and survey methodology. The questionnaire was pilot-tested for face validity and clarity in 10 physicians (LTC physicians and experts in osteoporosis management) identified by the investigators, whose recommended changes were incorporated into the final version.

The survey comprised of 11 items, including 3 open-ended questions (see Additional file 1). The survey questions presented to the participants aimed to address three major themes: 1) Are the recommendations put forth in the OC guidelines for fracture risk assessment (i.e. history, physical exam, biochemical evaluation, BMD testing and fracture risk assessment tools) applicable in the LTC setting? 2) What is your current approach to assessing fracture risk and therapeutic management of osteoporosis? 3) What are the barriers to optimal fracture prevention in LTC? Furthermore, included in the survey as closed-ended questions, three hypothetical clinical cases portraying LTC residents at high or moderate 10-year absolute risk for fragility fracture were presented to participants to evaluate their knowledge and understanding of the OC recommended fracture risk assessment approach.

### Analysis

Data analysis included descriptive statistics applied to the closed-ended questions, expressed as means (SD) for continuous variables and frequency (percentages) for categorical variables. Thematic Framework Analysis (TFA), a type of content analysis specially designed for qualitative data, was used to identify themes and subthemes in the data from the 3 open-ended questions [18,19]. We combined this data-driven inductive approach, letting the themes emerge from the data, plus a deductive approach, applying a template or codebook using the questions as the major heading or themes. We then ranked the themes in decreasing order of frequency as presented in the results section. The six stages of TFA we followed include: 1) familiarization (reading data to identify themes and ideas); 2) identify the thematic framework based on the literature, clinical experience of the team members and the data; 3) systematically apply the framework to all the data while updating the framework (indexing); 4) create displays of the data as matrices or tables to link data sources and themes (charting); 5) exploring patterns through comparing the data (mapping); and 6) developing explanations of the patterns found in the data (interpretation).

## Results

Three hundred and forty seven questionnaires were distributed and 87 (25%) participants submitted completed questionnaires.

Table 1 shows participants’ demographics; mean (SD) time in practice was 32 (11) years and most had over 50 LTC residents under their care. Eighty-seven percent of physicians considered prevention of osteoporosis-related fractures as important, and over 90% felt confident in their ability to assess fracture risk in their LTC residents.

**Table 1:** Physician demographics*

**Age, *mean (SD), years***	**59.6 (10.9)**
Male, *n (%)*	70.0 (81.4)
Time in practice, *mean (SD), years*	31.8 (11.4)
LTC residents, *mean (SD)*	102.6 (72.2)

*N = 88; response rate 25.4%.

### Applicability of the OC guidelines

**Figure 1:**
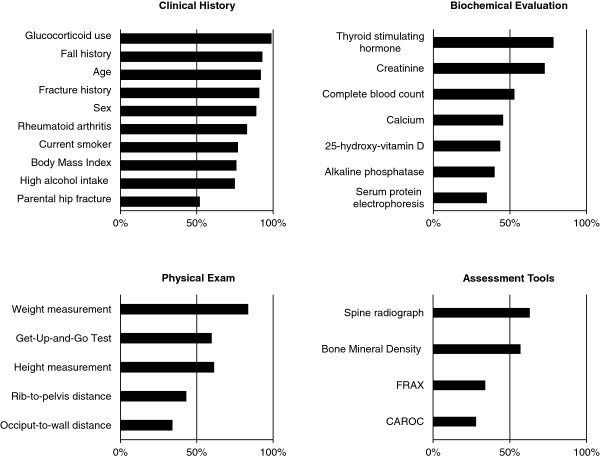
Physician-perceived applicability in LTC of fracture risk assessment tools recommended in the OC guidelines.

Figure 1 summarizes participants’ assessment of the applicability of the OC guidelines to the LTC population. Risk factors considered to be important on history and pertinent to document in the LTC setting included: glucocorticoid use, fall history, age, and the presence of previous fragility fractures. Recommended clinical evaluations considered applicable in LTC were the documentation of weight and height, Get Up and Go test, thyroid stimulating hormone (TSH) and serum creatinine levels. BMD measurement and spine radiographs were felt to be applicable in the LTC setting by approximately 55% of participants; the use of the validated fracture prediction tools FRAX and CAROC, as recommended by the OC guidelines (which include BMD and relevant clinical risk factors) were deemed applicable by less than 40%. Thirty-four percent of physicians said they actually used the FRAX or CAROC tools to evaluate fracture risk in LTC residents and 33% did not use any specific tools. Many reported that they considered all residents to be at high risk for fractures. Fracture risk assessment, an important first step to osteoporosis management, was reported by the participants as being performed by the LTC physicians (90%) or by nurses (56%) at the time of the residents’ admission.

### Clinical case scenarios

In response to the clinical case scenarios, over 85% of participants appropriately evaluated patients to be at high risk (over 20% probability of major osteoporotic fracture), whereas in the last case presenting a patient at moderate risk for fracture (between 10 and 20% probability of a major osteoporotic fractures), 54% provided the correct answer. The proportion of physicians who were uncertain of the hypothetical patient’s fracture risk was higher in the third case compared to the first two cases.

### Therapeutic options as recommended by OC guidelines

In terms of use of therapeutic options, recommended by the OC guidelines, the percentage of participants who reported that they “usually or always used” were: 70% for total calcium intake of 1200 mg daily, 94% for vitamin D 800 to 2000 IU daily, 53% for resistance training, 54% for core stability and 72% for balance training. If pharmacotherapy was felt to be indicated, bisphosphonates were the therapeutic class of pharmacological agents most often favoured (“occasionally-usually”: 91%), compared to denosumab (“occasionally-usually”: 24%), selective estrogen receptor modulators (“occasionally-usually”: 13%), calcitonin (“occasionally-usually”: 25%) and teriparatide (“never-seldom”: 93%).

### Perceived barriers to assessing fracture risk

Perceived barriers to assessing fracture risk were grouped under 4 themes: difficulty in acquiring necessary information to assess fracture risk (“impractical to obtain BMD or other tests in LTC”), resources constraints (“lack of manpower to apply fracture risk assessment tools” or “time constraints”), futility of fracture risk assessment (“unproven cost-benefit effectiveness of fracture risk assessment”, “short life expectancy of LTC residents”, “polypharmacy”) and other (“lack of collaboration from family members”) (Table 2).

**Table 2:** Physician-reported barriers* to fracture risk assessment or application of the OC guidelines in LTC, grouped in rank order by major theme

	**Rank**	**# of coded statements**
**Theme: Difficulty acquiring necessary information**	1	35
Lack of access to diagnostic testing		
Difficulty obtaining medical history		
**Theme: Resource constraints**	2	30
Time constraints		
Information-related issues		
Manpower/staff issues		
Financial constraints		
**Theme: Futility of fracture risk assessment**	3	28
Unproven cost-benefit effectiveness		
Polypharmacy		
Co-morbidities		
Lack of access to therapeutic tools		
**Theme: Other**	4	7
Resident or family cooperation		
Miscellaneous		

*100 statements from 59 respondents.

### Recommendations-facilitation

Participant responses to the questions on adaptations to the current OC and its relevance and applicability of fracture assessment in LTC were categorized along 3 themes. First, adapt tools and therapeutic recommendations (“remove need to perform BMD in fracture risk assessment”, “vitamin D should be recommended for everyone”); second make the guidelines relevant to LTC (“address lack of evidence in LTC population”, “presence of multimorbidities”), and third, other issues (Table 3).

**Table 3:** Physician-reported recommendations* to improve relevance and implementation of the OC guidelines in LTC, grouped in rank order by major theme

	**Rank**	**# of coded statements**
**Theme: Adapt tools and therapeutic recommendations**	1	34
Remove BMD from fracture risk assessment		
Adapt therapeutic recommendations		
Clarify fracture risk assessment recommendations		
Make format of guidelines and tools more user-friendly		
**Theme: Make guidelines relevant to LTC**	2	30
Address factors that make the LTC population unique		
Address short life expectancy of LTC residents		
Address lack of evidence in LTC population		
Address risk of therapeutic recommendations		
**Theme: Other**	3	3
Recommend the involvement of pharmacists		
Involve LTC physicians in guideline development		

*67 statements from 45 respondents.

To promote dissemination and uptake of the guidelines, continued access to educational sessions for all staff, and development of customizable data collection forms and of applications for smart phones and computers were frequently mentioned by respondents.

## Discussion

In this survey, physicians with a clinical practice that includes LTC residents report that the prevention of osteoporosis-related fractures is an important aspect of their practice; however the majority do not use OC recommendations for evaluation of fracture risk or osteoporosis management. LTC physicians report that they are confident in their ability to assess fracture risk, but almost half of them are not using the fracture risk assessment protocols recommended in the guidelines. Many respondents are aware of the OC guidelines’ recommendations and properly recognize patients at high risk of fractures, as demonstrated by their answers but they acknowledge a number of barriers to applying the recommendations in the LTC setting. There seem to be more uncertainty in identifying those who are at moderate risk who may not require pharmacotherapy. This inability to discern which resident actually might benefit from therapy may lead to under-treatment of this population. Furthermore, because of perceived barriers associated with using FRAX or CAROC, many LTC physicians may be adapting their own strategies for fracture risk assessment in LTC leading to suboptimal bone health management. Therefore, the responses collected in this survey support the need to establish the effectiveness of OC current guideline recommendations in the LTC population (for example the fracture prediction tools that require BMD measurements), to take into consideration the particular LTC setting and to enlist effective partnerships with clinicians, leaders and patient groups for future development and implementation of relevant best practice guidelines for this population.

Other work has also shown that a minority of physicians use recommended validated fracture prediction tools to assess fracture risk in their LTC residents. Difficulty accessing BMD and other evaluation modalities, unknown cost-benefit effectiveness of interventions in this population and lack of resources were the most often cited barriers to optimal fracture risk assessment. Our results mirror those obtained by McKercher et al. a decade ago in a survey of the members of the medical directors and advisors to LTC facilities in Ontario following the publication of the initial OC clinical guidelines in 1996 [15,20]. In their survey, 46% percent of respondents did not routinely assess fracture risk, 52% based their assessment on clinical factors only and 23% on BMD or spine radiographs. Perceived barriers to initiating treatment for osteoporosis included lack of access to BMD, unproven effectiveness of interventions in the LTC population, possible side effects of pharmacological treatments, time and cost of diagnosis and treatment, and patient reluctance. This situation is similar in many countries around the world [21,22]. In the United States, Colon-Emeric et al. surveyed LTC home administrators, physicians and nurses on the use of clinical practice guidelines in LTC [23]. The most frequently cited barriers to their implementation were provider concerns that guidelines were “checklists” to replace clinical judgment, limited facility resources, conflict with family representatives and facility policies that conflict with guidelines’ recommendations. A more recent survey also demonstrates the need for education and adaptation of osteoporosis guidelines of front-line staff in LTC in management of osteoporosis [24]. These results underscore the fact that guidelines targeted at community-dwelling men and women cannot be readily applied to those living in residential care, even if these guidelines are updated and broadly disseminated, as OC has done in 2010 [8]. Moving from evidence to practice in the clinical world requires integrated knowledge translation that include taking into consideration more than the knowledge to be transmitted but also the context or setting where the this will take place, the target audience and the facilitators (human resources and others) that will ensure changes in clinical practice can take place [10,25].

Nevertheless, there have been efforts to provide guidance for fracture prevention in residential care. A scoping review of strategies for the prevention of hip fractures in elderly nursing home residents documented that vitamin D supplementation and, in some cases, alendronate and hip protectors were associated with reduced fracture risk [26]. Consensus recommendations for fracture prevention in LTC have been published [27,28]; however, most recommendations are based on data obtained in clinical trials that excluded LTC residents, which may reduce physicians’ confidence in applying them [29]. Recently Rondondi et al. demonstrated that in an older population of LTC residents the 10-year fracture probability appeared to be mainly determined by age and clinical risk factors obtained by medical history, rather than by BMD or the presence of vertebral fractures on radiography [30], thereby supporting the concept that prediction rules in LTC may not necessitate evaluation of BMD or imaging. Additional barriers we have identified such as lack of resources to administer fracture assessment tools, resistance from family members, costs, polypharmacy, may help explain why LTC physicians do not initiate therapies in patients at high risk for fractures including those who have recently sustained a hip fracture [14,31,32]. There have been successful multifaceted interventions in LTC that have demonstrated reduced fracture rates associated with vitamin D supplementation and anti-osteoporosis therapy use in LTC [33,34]. Therefore, if barriers associated with applicability of treatment could be addressed or knowledge regarding interventions effective in LTC could be effectively translated into practice, the prevention of fractures in LTC could be optimized.

Our study is limited mostly by a modest response rate and the geographic limitation of the survey distribution (Ontario, Canada). Nevertheless, we feel that our questionnaire resulted in data consistent with those of previous surveys conducted in study populations in other jurisdictions, suggesting that our findings are translatable. It is possible that those that responded are physicians with an interest in osteoporosis, or who are more confident in their knowledge and practice around osteoporosis management. We are not able to confirm whether physician perceptions are consistent with practice. Like others, we have highlighted the urgent need to address barriers to fracture risk assessment in LTC with the development and dissemination of setting-specific best-evidence guidance with particular attention to the culture and nature of the environment and the identification of local champions.

## Conclusion

Osteoporosis-related fractures cause significant morbidity and loss of autonomy in the general population and even more strongly affect the frail elderly who live in LTC. To ensure optimal management of osteoporosis and prevent fractures in this population, evidence-based guidance applicable in the LTC setting must be developed and disseminated to physicians and front-line staff. We have shown that perceived barriers to fracture risk assessment and osteoporosis management in LTC have not changed over the last 10 years, contributing in part to the ongoing care gap in osteoporosis management. The results of this survey and of the published literature can greatly assist efforts by expert and community stakeholders to adapt consensus guidelines and bone health research priorities for use in the LTC setting in Canada. Such efforts are currently in progress.

## Competing interests

AP has received honoraria or grants from the following: Novartis Pharmaceuticals Canada, Eli Lilly Canada, Merck Frosst Canada, Amgen Canada, and Warner Chilcott Canada. LG has received a grant from Merck Frosst Canada. SNM has received honoraria or unrestricted research grants from the following: Amgen Canada, Novartis Pharmaceuticals Canada, Eli Lilly Canada and Merck Frosst Canada. All other authors have no financial disclosures.

## Authors’ contributions

MW: Participated in study design, data collection, statistical analysis, interpretation of results and drafted the initial manuscript with SNM. LL: Participated in study design, thematic-framework analysis, interpretation of results, and critically appraised review of the initial draft. SNM: Conceived the study, participated in study design, interpretation of results and drafted the initial manuscript with MW. LG, GI, CCK, AM and AP: Participated in study design, interpretation of results, and critically revised the manuscript for important intellectual content. All authors read and approved the final manuscript.

## Pre-publication history

The pre-publication history for this paper can be accessed here:

http://www.biomedcentral.com/1471-2318/13/109/prepub

## Supplementary Material

Additional file 1 **Survey.** Fracture risk assessment in long-term care. Click here for file
