# Supplementary material for: Fracture risk assessment in long-term care: a survey of long-term care physicians
Source: BMC Geriatr. 2013 Oct 18;13:109. doi: 10.1186/1471-2318-13-109 (PMC3853074; doi:10.1186/1471-2318-13-109)
Supplement: Additional file 1 — Survey. Fracture risk assessment in long-term care. [file 1471-2318-13-109-S1.pdf]

1. Prevention of fragility fracture in residents of LTC homes is an important aspect of my clinical practice.
   a. [Strongly Agree]
   b. [Agree]
   c. [Neutral]
   d. [Disagree]
   e. [Strongly Disagree]

2. My confidence in my ability to assess fracture risk of residents in LTC homes is:
   a. [Very Low]
   b. [Low]
   c. [Moderate]
   d. [High]
   e. [Very High]

3. Below is a summary of the clinical risk factors and assessment tools that the *2010 OC Guidelines* recommend using when assessing fracture risk. Please select whether you find each recommendation listed useful or applicable (Yes), or not useful or applicable (No), when assessing fracture risk of residents in LTC homes. If you have no opinion at this time please select "I don't know":

	Yes	No	I don't know
a. Clinical history (*risk factors*):			
1) Age	❒	❒	❒
2) Sex	❒	❒	❒
3) Body mass index	❒	❒	❒
4) Parental hip fracture	❒	❒	❒
5) Fragility fracture after age 40	❒	❒	❒
6) Falls in the previous 12 months	❒	❒	❒
7) Prolonged glucocorticoid use	❒	❒	❒
8) Rheumatoid arthritis	❒	❒	❒
9) Current smoker	❒	❒	❒
10) High alcohol intake (≥3 drinks per day)	❒	❒	❒
b. Physical exam:			
1) Weight measurement	❒	❒	❒
2) Height measurement	❒	❒	❒
3) Rib-to-pelvis distance	❒	❒	❒

4) Occiput-to-wall distance	❒	❒	❒
5) Get-Up-and-Go Test	❒	❒	❒
**c. Biochemical evaluation:**			
1) Calcium	❒	❒	❒
2) Complete blood count	❒	❒	❒
3) Creatinine	❒	❒	❒
4) Alkaline phosphatase	❒	❒	❒
5) Thyroid stimulating hormone	❒	❒	❒
6) Serum protein electrophoresis	❒	❒	❒
7) 25-hydroxy-vitamin D	❒	❒	❒
d. Bone Mineral Density testing	❒	❒	❒
e. Radiograph of the thoracic and lumbar spine	❒	❒	❒
f. Fracture assessment tools:			
1) CAROC			
a) Paper version	❒	❒	❒
b) App (smartphone)	❒	❒	❒
c) Computer or web-based	❒	❒	❒
2) FRAX			
a) Paper version	❒	❒	❒
b) App (smartphone)	❒	❒	❒
c) Computer or web-based	❒	❒	❒

4. Please indicate the frequency that you utilize each therapeutic option in your practice to reduce fracture risk of residents in your LTC home(s):

	Always	Usually	Occasionally	Seldom	Never
1) Calcium (through diet and supplements)					
a) < 1200mg daily	❒	❒	❒	❒	❒
b) 1200mg daily	❒	❒	❒	❒	❒
c) > 1200mg daily	❒	❒	❒	❒	❒

2) Vitamin D supplements					
a) < 800 IU daily	❐	❐	❐	❐	❐
b) 800-2000 IU daily	❐	❐	❐	❐	❐
c) > 2000 IU daily	❐	❐	❐	❐	❐
3) Exercises (or referral to exercise)					
a) Resistance training	❐	❐	❐	❐	❐
b) Core stability	❐	❐	❐	❐	❐
c) Balance	❐	❐	❐	❐	❐
4) Hip protectors	❐	❐	❐	❐	❐
5) Pharmacotherapy					
a) Bisphosphonates (ex. Didrocal, Fosomax, Fosavance, Actonel, Aclasta)	❐	❐	❐	❐	❐
b) RANK ligand inhibitor (ex. Prolia)	❐	❐	❐	❐	❐
c) Selective estrogen receptor modulators (ex. Evista)	❐	❐	❐	❐	❐
d) Hormone therapy	❐	❐	❐	❐	❐
e) Calcitonin (ex. Miacalcin)	❐	❐	❐	❐	❐
f) Bone-forming agents (ex. Forteo)	❐	❐	❐	❐	❐

5. Who currently assesses fracture risk of residents in your LTC home(s)? (*Please select all that apply*)

a. Physician	❐
b. Physician Assistant	❐
c. Nurse	❐
d. Nurse Practitioner	❐
e. Physiotherapist	❐
f. Pharmacist	❐
g. No one	❐
h. I don't know	❐

i. Other (*please specify*)___________________

6. What tool(s), if any, do you use to evaluate fracture risk of residents in your LTC home(s)? (*Please select all that apply*)
   a. FRAX ❐
   b. CAROC ❐
   c. None ❐
   d. I don't know ❐
   e. Other (*please specify*)___________________

7. What are the barriers to assessing fracture risk of residents in LTC homes or in the application of the *2010 OC Guidelines*? *(If you have no opinions please state, "No opinion at this time".)*

   ____
   ____
   ____

8. We would like to assess your familiarity with fracture risk assessment in a clinical setting. For each scenario below, please determine the absolute 10-year risk of fracture:

   a. Scenario #1: An 85 year-old man has just been transferred from acute care for a hip fracture to your LTC home. He has never received treatment for osteoporosis prior to admission. What is his absolute risk of fracture over the next 10 years?
      1) [Low]
      2) [Moderate]
      3) [High]
      4) [Not sure]

   b. Scenario #2: A 75 year-old woman is admitted to your LTC home. She has moderate dementia and hypertension, walks with a walker and has not sustained any falls in the last year. You learn that she broke her left wrist at age 65 and her left proximal humerus 2 years ago. Since then she has been using a walker. What is her absolute risk of fracture over the next 10 years?
      1) [Low]
      2) [Moderate]
      3) [High]
      4) [Not sure]

c. Scenario #3: An 80 year-old woman's BMD T scores are -2.6 at the lumbar spine and -2.0 at the femoral neck. She denies previous fractures or glucocorticoid use in the past. What is her absolute risk of fracture over the next 10 years?
   1) [Low]
   2) [Moderate]
   3) [High]
   4) [Not sure]

9. What do you think can be done to improve LTC physicians' knowledge of the *2010 OC Guidelines' recommendations*? *(If you have no opinions please state, "No opinion at this time".)*

   ____
   ____
   ____

10. What adaptations would you like to see made to the *2010 OC Guidelines* for the recommendations to be more relevant and useful for the practicing LTC physicians? *(If you have no opinions please state, "No opinion at this time".)*

   ____
   ____
   ____

11. The final set of questions is for demographic purposes only. Remember responses are anonymous and cannot be tracked. Please indicate:

a. Your age:
   1) [30-35]
   2) […]
   3) [80-85]
   4) Prefer not to say

b. Your gender:
   1) [Male]
   2) [Female]
   3) Prefer not to say

c. The number of years you have been in practice:
   1) [1-5]
   2) [……]
   3) [51-55]

4) Prefer not to say

d. The number of years you have been working in the LTC environment:
1) [1-5]
2) [……]
3) [51-55]
4) Prefer not to say

e. The number of residents in LTC homes currently under your care:
1) [1-50]
2) [……]
3) [250-300]
4) [>300]
5) Prefer not to say
